# Supplementary material for: Angelica sinensis polysaccharide as potential protectants against recurrent spontaneous abortion: focus on autophagy regulation
Source: Front Med (Lausanne). 2025 Jan 15;12:1522503. doi: 10.3389/fmed.2025.1522503 (PMC11774876; doi:10.3389/fmed.2025.1522503)
Supplement: Supplementary material S1 — The main instruments used during the LC-MS process, along with their models/specifications and manufacturers. [file Supplementary_file_1.zip › Metabolomics sequencing data FC1.2/常用软件及使用说明.html]

分析软件.knit


# **常用软件说明及使用文档**

# 1 欧易云平台连接

欧易云平台提供多种在线可视化分析内容,以下是欧易云平台的访问连接:

https://cloud.oebiotech.cn/task/

# 2 定性软件

## 2.1 格式转换软件-AbfConverter.4.0.0

AbfConverter：通常采集到的质谱原始数据有各种各样的数据格式，使用AbfConverter软件进行数据格式的转换，将质谱原始数据转换成abf格式的数据以便用MS-DIAL进行后续的定性处理！

## 2.2 定性软件-MS-DIAL软件

MS-DIAL是一款针对非靶向代谢组学的数据处理的第三方软件，最大的优势在于能够兼容来自不同仪器（Agilent，Bruker，LECO，Sciex， Shimadzu，Thermo和Waters）获得的各类质谱数据（GC-MS，GC-MS/MS，LC-MS和LC-MS/MS）。此外像netCDF（AIA）和mzML等通用的数据格式也可以被软件识别。

## 2.3 定性软件-Progenesis QI软件(收费软件)

Progenesis Progenesis QI是Waters公司开发的独立数据分析平台，可支持沃特世、赛默飞世尔、Sciex、安捷伦和布鲁克公司的LC-MS数据分析。同时也支持通用格式的数据，例如.mzML、.mzXML和NetCDF等。Progenesis QI可以精确定量和识别样品中变化明显的化合物，具有高度直观的菜单引导的工作流程，能快速、客观、可靠地发现感兴趣的化合物。

# 3 分析软件

## 3.1 R软件

R是一套完整的数据处理、计算和制图软件系统。其功能包括：数据存储和处理系统,数组运算工具（其向量、矩阵运算方面功能尤其强大）,完整连贯的统计分析工具,优秀的统计制图功能,简便而强大的编程语言：可操纵数据的输入和输出,可实现分支、循环,用户可自定义功能。

RStudio官网：https://www.rstudio.com/

R与RStudio下载参考地址： https://blog.csdn.net/devoteto/article/details/110854875

## 3.2 文本编辑器-Notepad++

Notepad++软件：可以用来编写及修改R语言代码等,比Windows默认的notepad功能更加强大,推荐使用

# 4 拓展软件

## 4.1 AI软件与PS软件

两个可以对图片进行修改、编辑等操作的软件

# 5 AI软件使用说明

## 5.1 打开AI软件

1.可以看到图片上所展示的画板及工具箱等页面

2.打开要编辑的pdf图片文件

例如:我们要对下面这张图片进行修改

## 5.2 编辑过程

我们的修改要求如下：

(1).修改图片中的配色

(2).修改图片示例文字为中文

(3).修改图片中数据标签的字体大小为12pt

(4).修改图片中数据标签的字体样式为Airal

(5).修改图片的数据标签位置将其移至圆形内部

1.首先修改图片中的配色

具体步骤如图：导入颜色块→选中需要修改颜色的部分→点击拾色器→点击颜色块

2.将图例中英文文字修改为中文

具体步骤如图：鼠标右键选择需要修改的文字，将其替换为中文

3.修改数据标签字体为12pt

具体步骤如图：鼠标右键选中所有需要修改的文字→菜单栏中选择文字→选择大小→选择12 pt

4.修改数据标签字体为Airal

具体步骤如图：鼠标右键选中所有需要修改的文字→菜单栏中选择文字→选择字体→选择Airal

5.修改数据标签的位置

具体步骤如图：鼠标右键选中需要修改的文字→移动至圆内的位置

## 5.3 导出文件

最后,我们修改好之后就可以对其进行保存,AI软件导出图片不仅可以导出pdf格式的,也可以选择其他格式。

1.具体步骤如图：在菜单栏中选择文件→根据需要保存的格式选择存储为或导出为→选择保存类型→保存或导出

2.修改好的pdf图片

下面就是我们最终修改好的图片
